# Pilot Study of Trace Elements in the Infusion of Medicinal Plants Used for Diabetes Treatment

**DOI:** 10.1155/2022/3021396

**Published:** 2022-04-18

**Authors:** Eid I. Brima, Saifeldin M. Siddeeg

**Affiliations:** ^1^Department of Chemistry, College of Science, King Khalid University, Abha 61413, Saudi Arabia; ^2^School of Allied Health Sciences, Faculty of Health and Life Science, De Montfort University, Leicester LE1 9BH, UK; ^3^Department of Chemistry, School of Natural Sciences, Faculty of Science and Engineering, The University of Manchester, Manchester, M13 9PL, UK

## Abstract

The purpose of this study was to evaluate trace element contents in different medicinal plants used for diabetes treatments by residents in Asir region. Five medicinal plants—Tut leaves (*Mulberry*), olive leaves (*Olea europaea*), clove (*Syzygium aromaticum*), Luban Dhakar (*Boswellia carterii*), and Karela or bitter melon (*Momordica charantia*)—were collected from two cities, Khamis Mushait and Abha, in the Asir region, Saudi Arabia. Infusions (hot water extracts) were obtained from each plant, and elemental analysis was conducted using inductively coupled plasma mass spectrometry (ICP-MS). Fourteen elements (Al, Cr, Mn, Fe, Co, Ni, Cu, Zn, As, Se, Sr, Cd, Ba, and Pb) were measured in all plants. The results revealed that the fourteen elements were present at different levels (µg/g) in all plants. *Momordica charantia* exhibited high levels of essential (Mn (251.4), Co (1.18), Cu (54.64), and Se (2.18)) and toxic elements (Al (39.20), As (0.57), Cd (0.33), and Pb (4.48)), followed by *Syzygium aromaticum* (Mn (736.36)) and *Boswellia carterii* (Pb (0.93)), which exceeded the PMTDI in traditional doses used for diabetes treatments. However, *Mulberry* and *Olea europaea* did not exceed the daily guideline values for all elements. Based on our findings, we cautiously recommend the latter two plants for the traditional treatment of diabetes, because they are not considered as source of harm based on their levels of elements. Their use should be restricted by comprehensive compound analysis to guarantee their safe use.

## 1. Introduction

The existence of many essential human nutrients, including elements in plants, enables them to significantly affect human health. It is known that the excess or deficiency of essential elements critically affects biochemical processes within the human body. The amount of elements varies among plants; thus, plants with considerable content are termed ‘medicinal plants.' Medicinal plants are used as starting materials to produce medications. In addition, they can be used directly prior to any chemical therapy to treat certain diseases [1]. This direct usage of fresh plants has a long history in human medicine. It has been reported that 70–80% of the global population use medicinal plants as an alternative medicine to treat some types of diseases [2]. Therefore, medicinal plants are important for pharmacological research and drug production, and many diseases are treated with alternative medicine today, especially in areas enriched with medicinal plants.

One of the major and growing contemporary health concerns is diabetes mellitus (DM). The figures of affected people are widely debated; however, according to World Health Organization (WHO) data, the disease affects about 200 million of the world's population. In Saudi Arabia, a recent study concluded that about 30% of the population were afflicted with diabetes [3]; hence, the disease represents a global as well as a local health problem. The disease is associated with unnatural levels of glucose in the blood, as a result of the unusual function of beta cells in the pancreas [4]. Glucose levels in the blood are controlled through the hormone insulin. Therefore, the deficiency of insulin and/or the ineffectiveness of the insulin produced are the main causes that disturb the metabolic process in diabetes; this results in the sugar present in the blood [5]. It has been claimed that deviations in the metabolism of some trace elements, such as Cu, Zn, Mg, and Mn, are associated with diabetes [6]. Researchers have concluded that some trace elements, such as copper, zinc, selenium, and manganese, may help in protecting the insulin-secreting pancreatic *β*-cells [7]. Therefore, the quantification of trace elements in medicinal plants used by local diabetic people can provide useful information about the safety of utilization and the effectiveness of such herbal medicine.

In the present study, fourteen trace elements concentrations were determined in five antidiabetic medicinal plants used by local diabetic residents in the Asir region using the inductively coupled plasma mass spectrometry (ICP-MS) technique.

The purpose of this study was to determine both essential and toxic trace elements in five antidiabetic medicinal plants. Additionally, they were evaluated as sources of both types of elements, and the efficacy of their use was based on elemental analysis.

## 2. Methods

### 2.1. Samples' Collection and Pretreatment

Five types of medicinal plants were collected from local markets (Attar's shops) in the cities of Khamis Mushait and Abha, Saudi Arabia. These medicinal plants are traditionally used for diabetic treatments. The types of medicinal plants sampled were Tut leaves (*Mulberry*), olive leaves (*Olea europaea*), clove (*Syzygium aromaticum*), Luban Dhakar (*Boswellia carterii*), and Karela or bitter melon (*Momordica charantia*). In total, nine samples were collected and pretreated for elemental measurements. All plants are presented in Table 1.

### 2.2. Infusion of the Samples

One gram from each plant type was boiled in deionized water for 10 minutes and made up to 50 ml. The infusion (filtrate solution) was filtered with a syringe through a 0.45 µm filter millipore before elemental analysis. Only Karela was dried in an oven overnight at 70°C and then powdered, before following the same procedure. This is because Karela's samples were wet, while other plants' samples were dry.

### 2.3. Chemicals and Reagents

A single-stock solution mixture of 27 elements at a concentration of 10 µg/mL (Agilent Technologies, USA) was used for standards preparation. Calibration standards 2, 10, 20, 40, and 100 µg/L were prepared by dilution with 1% HNO_3_.

### 2.4. Elemental Analysis Using Inductively Coupled Plasma Mass Spectrometry (ICP-MS)

In total, fourteen elements were measured in all collected samples. Concentrations of the fourteen elements—aluminum (Al), chromium (Cr), manganese (Mn), iron (Fe), cobalt (Co), nickel (Ni), copper (Cu), zinc (Zn), arsenic (As), selenium (Se), strontium (Sr), cadmium (Cd), barium (Ba), and lead (Pb)—were measured in each sample by Agilent 7900 ICP-MS at De Montfort University, United Kingdom. Samples were divided into two parts and analyzed in duplicate (*n* = 4). Oxide ratios and double-charge ratios were 2.5% and 1.1%, respectively. The Agilent 7900 ICP-MS operating conditions are presented in Table 2.

### 2.5. Quality Control

A method was validated for all measured elements; limits of detection (LODs) and limits of quantification (LOQs) were calculated. LOD = Xb1 +3Sb1 and LOQ = Xb1 + 10Sb1, where Xb1 is the mean concentration of the blank and Sb1 is the standard deviation of the blank (deionized water). LODs and LOQs (expressed in µg/L) for the measured elements were Al (6.24 and 20.8), Cr (0.09 and 0.3), Mn (1.26 and 4.2), Fe (15.1 and 49.5), Co (0.04 and 0.11), Ni (0.38 and 1.15), Cu (1.05 and 3.5), Zn (1.38 and 4.6), As (0.17 and 0.45), Se (0.27 and 0.9), Sr (3.54 and 11.8), Cd (0.03 and 0.08), Ba (2.31 and 7.7), and Pb (0.66 and 2.2).

The LODs and LOQs for elements were treated the same as samples ((µg/L *∗* 0.05 L)/1g). Therefore, the LODs and LOQs for the real samples (expressed in µg/g) are as follows: Al (0.312 and 1.04), Cr (0.005 and 0.015), Mn (0.063 and 0.21), Fe (0.755 and 2.475), Co (0.002 and 0.006), Ni (0.019 and 0.058), Cu (0.053 and 0.175), Zn (0.069 and 0.23), As (0.009 and 0.023), Se (0.014 and 0.045), Sr (0.177 and 0.59), Cd (0.002 and 0.004), Ba (0.116 and 0.385), and Pb (0.033 and 0.11).

Quality control checks were performed by measuring 50 µg/L of mixed elements three times after each batch of five samples. Recoveries (%) of triplicate measurements of each element were as follows: Al (92.7), Cr (103.7), Mn (102.4), Fe (115.9), Co (107.6), Ni (110.3), Cu (108.6), Zn (149.2), As (125.7), Se (107.8), Sr (115.3), Cd (117.2), Ba (131.6), and Pb (115.5). High recoveries revealed from Zn, As, and Ba are not surprising, since measurements were spread over 15 samples (3x after each batch of 5 samples).

### 2.6. Quality Assurance

A 50 µg/L mixed standard was spiked in a sample and the obtained recoveries (%) were as follows: Al (93.3), Cr (92.4), Mn (92.9), Fe (85.6), Co (94.4), Ni (92.8), Cu (108.6), Zn (95.3), As (97.9), Se (96.5), Sr (93.7), Cd (93.6), Ba (95.8), and Pb (93.4). Actually, we had no CRM to be used to assess the accuracy of the method, specifically for infusion method. Therefore, recoveries range between 93 and 109% are used instead.

### 2.7. Statistical Analysis

Statistical Package for the Social Sciences (SPSS), version 20, was used for statistical analysis. Differences in the concentrations of elements among the medicinal plants were evaluated by using ANOVA (SPSS) with a 95% confidence level.

## 3. Results

Average concentrations (µg/g) of the fourteen measured elements (Al, Cr, Mn, Fe, Co, Ni, Cu, Zn, As, Se, Sr, Cd, Ba, and Pb) in the five types of medicinal plant (Karela, clove, Luban Dhakar, olive leaves, and Tut leaves) are presented in Table 3.

A dose weight was calculated based on personal communications with consumers. The average weights (g) of three bunches in five fingers were as follows: Tut leaves (2.37), clove (1.98), olive leaves (4.04), and Luban Dhakar (7.80). Karela was calculated as an average of three pieces (dry weight = 9.22; wet weight = 164.72 g, with moisture = 94.4%). Exposure calculations should be based on the actual dose size and frequency of use. Diabetic patients who drink the infusion of each plant are exposed to different quantities (µg) of elements included in the specific plant. We considered the number of grams in one dose of each plant as mentioned above. The exposures (µg) to each element related to the specific plant are presented in Table 4. The frequency of use was considered as one.

**Figure 1:**
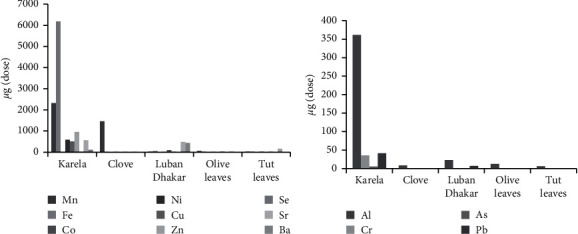
Amount (µg) of each element in each dose of a plant: (a) essential elements and (b) toxic elements.

For comparison purposes, elements were divided into essential and toxic elements as explained in Figure 1(a) for essential elements and Figure 1(b) for toxic elements. Karela showed the highest contents for both essential and toxic elements.

SPSS was used to explore correlations between the medicinal plants related to the contents of the measured elements. The correlations are presented in Table 5.

## 4. Discussion

The elemental contents of the five medicinal plants in their infusions are presented in Table 3. The infusion was prepared from one gram of each plant. Karela exhibited the highest levels for the toxic elements (As and Pb), exceeding PMTDI values [9, 14]. The trends of decreasing element concentrations in the five medicinal plants are as follows: Karela Fe > Mn > Zn > Ni > Sr > Cu > Al > Ba > Pb > Cr > Se > Co > As > Cd; clove Mn > Zn > Sr > Ni > Fe > Al > Ba > Cu > Pb > Se > Cr ≥ As > Co > Cd; Luban Dhakar Sr > Ba > Cu > Fe > Mn > Al > Zn > Ni > Pb > Se > Cr > As > Co ≥ Cd; olive leaves Mn > Zn > Fe > Ni > Sr > Al > Ni > Cu > Ba > Pb > Se > Co ≥ Cr > Cd; Tut leaves Sr > Mn > Zn > Ni > Fe > Ba > Al > Cu > Se > Pb > As > Co > Cr > Cd.

In general, all five medicinal plants exhibited cadmium at the lowest levels. However, iron, manganese, and strontium had the highest concentrations in Karela, clove and olive leaves, and Luban Dhakar and Tut leaves, respectively. The element concentrations in clove represented a similar trend to that of olive leaves. Additionally, Luban Dhakar and Tut leaves displayed similar trends. The correlations between the medicinal plants related to their elements contents were confirmed statistically using SPSS, and the results are presented in Table 5. Significant correlations were recorded between clove and olive leaves; additionally, Luban Dhakar and Tut leaves were also correlated. The element contents of the clove showed a highly significant correlation (*p* < 0.01) with the element contents of olive leaves. Additionally, Luban Dhakar and Tut leaves exhibited a similar correlation (0.694) significant at *p* = 0.01 as shown in Table 5. Karela was not correlated with any of the other plants. This is because Karela is a source of more essential and toxic elements, which gave it a unique profile related to element concentrations.

Dose exposures presented in Table 4 are based on one dose from each plant. The results showed that Karela exceeded PMTDI values [10, 12, 13, 13] for essential elements (Mn, Co, Cu, and Se) and PMTDI values [8, 9, 14, 17] for toxic elements (Al, As, Cd, and Pb). We advise against the use of these plants (Karela and Luban Dhakar), because they are sources of toxic elements, specifically PB, which exceeded the PMTDI. A dose of Luban Dhakar exceeded the PMTDI of Pb > 2-fold, while for Karela it was >11-fold [9].

We infer that Karela is a natural accumulator of both essential and toxic elements. Therefore, Karela is considered a major source of exposure to both types of elements. One dose of Karela contained high percentages of Al (120%), Mn (644%), Co (73%), Cu (101%), As (1252%), Se (214%), Cd (304%), and Pb (1157%), calculated against the PMTDI. Therefore, an infusion of Karela contains high concentrations of essential and toxic elements, which lead to health harm if used for treatment. A recent study suggested the use of Karela for diabetic treatment due to the high Zn contents [20]. However, the limitation of this study was that it was not comprehensive and only focused on the investigation of one essential element. The study did not investigate the contents of toxic elements in Karela, which cause adverse health effects in humans. Holistic studies including both types of elements are always required to ensure the safe use of such medicinal plants, regarding the levels of toxic metals [21]. Notably, high levels of both essential and toxic elements can cause negative health implications, such as mutagenic effects in humans, reduced growth, morphological abnormalities, and increased mortality [22]. Therefore, paper [20] is suitable for pharmacological research, to extract and use specific protein fractions of the plant. However, their study approach is only suitable for conventional uses of medicinal plants, whereas diabetic patients use the whole plant.

Our results showed that one dose (1.98 g) of ground clove contributed to 66.3% of Mn of the PMTDI. This agrees with other reports, which have stated that this plant contributes to 55–63% of the daily value (DV) in one teaspoon (≥2 g) of ground cloves [23, 24].

Many studies have suggested that Mn, Cu, Zn, and Cr are responsible for the secretion of insulin from the beta cells of the islets of Langerhans and are involved in increasing the ability of insulin action [25, 26].

Mn was found in high concentrations in Karela and clove (Table 3). This requires further investigation; as in a previous study [27], it is indicated that manganese plays a role in immunity through the regulation of blood sugar and cell energy. In addition, Mn is essential for the synthesis and secretion of insulin, and a lack of this element may reduce insulin discharge and alter carbohydrate and lipid metabolism [28]. The contents of Mn in the five medicinal plants in decreasing order were as follows: clove > Karela > olive leaves > Tut leaves > Luban Dhakar (Table 3).

Zinc, copper, and chromium play significant roles, along with calcium and manganese, in glucose tolerance factor (GTF), which affects the level of glucose in the blood by regulating insulin [29]. Interestingly, the levels of Zn in olive leaves and cloves were about the same, whereas Tut leaves presented the highest and Luban Dhakar presented the lowest levels. The plant contents of elemental copper were similar for clove, olive leaves, and Tut leaves, with Karela presenting the highest levels, followed by Luban Dhakar, as shown in Table 4. For chromium, the highest concentration was found in Karela, which was 50–80-fold higher than Cr contents in the other four plants, all of which had similar concentrations (Table 4). Therefore, our results showed that the highest concentration of Cr was in the Karela infusion, which is promising for it to be suitable for diabetes treatment, due to the role of Cr in reducing glycated hemoglobin (HbA1c) in type 2 diabetic patients [30]. A recent study encouraged the use of Karela as an antidiabetic treatment due to high Cr contents [31]. Moreover, based on their speciation analysis, they reported that high Cr contents are bound to protein fractions. However, more speciation analysis is needed to determine which species (Cr (III) or Cr (VI)) of Cr is most present in Karela's protein fractions. This is because exposure to Cr (VI) poses a toxic risk to human health [32], whereas Cr (III) is essential for human health, improving glucose tolerance [33].

Elemental selenium is a well-known antioxidant and thus may help in reducing the health risks associated with insulin irregularities [34]. Selenium content was found to be high in Karela, up to 22-fold greater than the other four plant contents. However, the concentration of Se in all plants was lower than the guidelines set by the WHO. It has been reported experimentally that some Se compounds behave similarly to insulin, demonstrating a positive link between higher selenium levels and the prevalence of diabetes [35, 36]. A recent study investigated the effects of Se supplements on glucose homeostasis. The study concluded that Se might affect the control of blood sugar at different levels of regulation, associated with insulin signaling, glycolysis, and pyruvate metabolism [37]. Jablonska et al. concluded their study with an open question; the exact effects of selenium supplementation on glucose homeostasis and diabetes risk remain unclear.

The presence of large quantities of toxic elements (As, Al, Cd, and Pb) in Karela and Luban Dhakar exceeded the PMTDI, the allowance level recommended by the WHO (Table 4). These high levels of toxic elements represent a serious health risk for patients who use these plants for diabetic treatment. It has been reported that heavy metals such as lead, arsenic, and cadmium contribute toward the binding of *β*-cells in the pancreas and thus stimulate an autoimmune reaction to *β*-cells through the actual destruction of *β*-cells. In particular, lead is bonded to proteins that could induce morphological changes in the body [29]. A previous study [38] reported a significant positive correlation between blood lead concentrations and fasting blood glucose, which led them to infer that there is a possible association between Pb exposure and diabetes. A recent study [39] investigated the association between arsenic exposure and diabetes; the authors concluded that exposure to arsenic was linked to the development of diabetes. Another study [40] suggested that exposure to arsenic causes oxidative stress, which, in turn, causes *β*-cell dysfunction and glucose homeostasis, and then induces diabetes, although this remains a hypothesis. It was found that aluminum toxicity causes adverse diabetic effects, in a study that investigated the effects of diabetes mellitus and aluminum toxicity [41]. A previous study [42] investigated the effect of aluminum exposure on rats' testicular tissue. This study concluded that the exposure to aluminum exacerbated diabetes-induced testicular lesions and impaired male reproductive variables in Wistar rats. A recent study [43] investigated the effects of cadmium exposure in rats and concluded that Cd exposure might increase the risk of diabetes. It was also inferred that exposure to cadmium decreased the activity of liver glucokinase, which affects sugar regulation. In a review article [44], groups of studies demonstrated that exposure to Cd affects adipose tissue, which leads to insulin resistance and enhances diabetes.

We measured two elements (Ba and Sr) considered as nonessential in medicinal plants. However, strontium is essential for bone metabolism [45]. Our results showed that Ba exceeded the PMTDI (200 ug) set by the Scientific Committee on Health and Environmental Risks [46] in Luban Dhakar (Table 4). Barium salts (BaSO_4_ and BaCO_3_) have a role in diabetes therapy. As concluded by a very recent study [47], an in vivo study on rats has proven the effectiveness of barium salts in controlling hyperglycemia after oral ingestion. This leads us to conclude that Luban Dhakar is an effective traditional treatment for diabetes based on its high Ba content, which could be used to manage blood sugar levels. Another study [48] concluded that plasma strontium was inversely associated with diabetes and impaired glucose regulation. Their study was yet to explore the mechanism which linked high Sr contents with low blood glucose levels in diabetic patients. Another study showed that strontium has antidiabetic effects by reducing blood glucose levels and improving the tolerance to insulin in diabetic mice [49]. Based on the conclusions of these studies, we can infer that Ba and Sr can be considered for diabetes treatment.

In fact, a comprehensive analysis is needed, including compound and speciation analyses, to guarantee the safety of the global community using these medicinal plants. Our conclusion is based on an elemental analysis point of view; however, more research is required to confirm our findings and to enhance the understanding of the health benefits and harmful effects of these medicinal plants. We recommend that future studies investigating such plants should cultivate them in clean soil supplied with water free from contamination (i.e., a controlled growth area) to ensure that results are not influenced by external parameters.

## 5. Conclusion

This study measured fourteen elements, including essential, nonessential, and toxic elements, in five medicinal plants. In a traditional dose of these plants for diabetes treatment, some plants exhibited high concentrations of some elements, exceeding the PMDTI. Karela displayed the highest contents for both essential (Mn, Co, Cu, and Se) and toxic elements (Al, As, Cd, and Pb), followed by clove for Mn and Luban Dhakar for Pb. Therefore, we advise against the use of Karela, clove, and Luban Dhakar as traditional antidiabetic treatments. However, we cautiously recommend the use of the two other medicinal plants (olive leaves and Tut leaves) based on their low levels of essential and toxic elements, which did not exceed the guideline values. Further studies, including compound analysis, are required for these medicinal plants to confirm the safety of the continued traditional usage of these plants for diabetes treatment. Also for future studies, the physiochemical properties of soil where these plants grow such as soil pH and organic matter will be of interest to assess and their extent to uptake from soil.

## Figures and Tables

**Table 1:** The studied plant species and the parts used for both diabetes treatment and analysis.

Scientific name	Common name	Used part
*Mulberry*	Tut	Leaves
*Olea europaea*	Olive	Leaves
*Syzygium aromaticum*	Clove	Seeds
*Boswellia carterii*	Luban Dhakar	Gum
*Momordica charantia*	Karela	Fruit

**Table 2:** Operating conditions for the Agilent 7900 ICP-MS.

Rf power	1550 W
Sampling depth	8 mm
Carrier gas	1.05 L/min
Nebulizer pump	0.1 rps
Integration time	0.1 s
Sampling period	0.311 s
Acquisition time	22.74 s
Cell gas	He

**Table 3:** Average concentrations (µg/g) of fourteen measured elements in five types of medicinal plants, mean ± SD (*n* = 4) with a 95% confidence level. The infusion was one gram of each plant boiled in deionized water for ten minutes.

Element	Karela	Clove	Luban Dhakar	Olive leaves	Tut leaves
Al	39.18 ± 1.27	4.21 ± 0.34	2.94 ± 2.13	3.13 ± 1.50	2.73 ± 0.85
Cr	3.85 ± 0.22	0.05 ± 0.01	0.08 ± 0.00	0.06 ± 0.01	0.07 ± 0.02
Mn	251.40 ± 8.41	736.36 ± 40.42	3.13 ± 0.22	14.44 ± 2.64	12.20 ± 2.27
Fe	671.13 ± 11.02	4.26 ± 0.15	6.98 ± 0.03	4.87 ± 1.93	6.64 ± 1.39
Co	1.20 ± 0.02	0.03 ± 0.00	0.02 ± 0.00	0.06 ± 0.05	0.08 ± 0.03
Ni	64.39 ± 4.63	4.25 ± 0.39	1.62 ± 0.12	3.06 ± 1.24	7.31 ± 2.12
Cu	54.64 ± 4.28	3.55 ± 0.22	12.70 ± 0.53	1.65 ± 0.12	2.55 ± 0.40
Zn	103.78 ± 7.59	5.97 ± 4.49	2.30 ± 1.74	5.80 ± 3.94	9.09 ± 6.68
As	0.57 ± 0.02	0.05 ± 0.01	0.04 ± 0.01	0.04 ± 0.01	0.12 ± 0.05
Se	2.18 ± 0.16	0.11 ± 0.01	0.09 ± 0.05	0.12 ± 0.07	0.21 ± 0.03
Sr	60.97 ± 1.44	4.74 ± 0.06	61.72 ± 4.25	3.85 ± 1.30	67.39 ± 5.86
Cd	0.33 ± 0.01	< LOD	0.02 ± 0.01	0.01 ± 0.01	< LOD
Ba	13.10 ± 0.32	3.57 ± 0.23	56.95 ± 1.37	1.25 ± 0.38	3.38 ± 0.92
Pb	4.48 ± 0.02	0.36 ± 0.05	0.93 ± 0.01	0.15 ± 0.03	0.20 ± 0.01

**Table 4:** Exposure (µg) to each element related to a specific plant, based on the number of grams used in each dose of a plant. Provisional maximum tolerable daily intake (PMTDI).

Element	Karela	Clove	Luban Dhakar	Olive leaves	Tut leaves	PMTDI (µg/day)	Reference
Al	361.27 ± 9.90	8.33 ± 0.68	22.97 ± 16.59	12.63 ± 11.74	6.48 ± 2.02	143–7000	[8]
Cr	35.53 ± 1.71	0.10 ± 0.03	0.59 ± 0.04	0.23 ± 0.06	0.17 ± 0.04	100	[9]
Mn	2317.94 ± 65.63	1458.00 ± 80.04	24.38 ± 1.75	58.35 ± 20.62	28.91 ± 5.39	1800–2200	[10]
Fe	6187.86 ± 85.99	8.44 ± 0.30	54.45 ± 0.25	19.66 ± 15.08	15.74 ± 3.29	11000	[11]
Co	11.04 ± 0.17	0.06 ± 0.01	0.16 ± 0.01	0.24 ± 0.35	0.19 ± 0.08	5–8	[12]
Ni	593.68 ± 36.15	8.41 ± 0.76	12.62 ± 0.97	12.38 ± 9.65	17.33 ± 5.04	1000	[10]
Cu	503.78 ± 33.40	7.02 ± 0.43	99.07 ± 4.16	6.68 ± 0.96	6.04 ± 0.95	500	[13]
Zn	956.81 ± 59.21	11.82 ± 8.90	17.98 ± 13.57	23.43 ± 30.73	21.54 ± 15.83	1000	[13]
As	5.26 ± 0.18	0.09 ± 0.02	0.28 ± 0.06	0.18 ± 0.06	0.28 ± 0.12	0.42	[14]
Se	20.12 ± 1.23	0.21 ± 0.03	0.68 ± 0.36	0.47 ± 0.55	0.49 ± 0.08	70	[15]
Sr	562.17 ± 11.23	9.38 ± 0.13	481.43 ± 33.19	15.57 ± 10.13	159.72 ± 13.88	1900	[16]
Cd	3.04 ± 0.08	0.00 ± 0.00	0.13 ± 0.06	0.03 ± 0.08	0.01 ± 0.01	1	[17]
Ba	120.80 ± 2.47	7.07 ± 0.46	444.24 ± 10.67	5.06 ± 2.99	8.00 ± 2.19	750 (200)	[18, 19]
Pb	41.31 ± 0.17	0.70 ± 0.11	7.25 ± 0.07	0.60 ± 0.27	0.48 ± 0.02	3.57	[9]

**Table 5:** Correlations between the five different medicinal plants associated with the fourteen measured elements.

	Karela	Olive leaves	Tut leaves	Clove
Olive leaves	0.510			
Tut leaves	0.077	0.283		
Clove	0.261	0.867*∗∗*	0.074	
Luban Dhakar	−0.050	0.024	0.694*∗∗*	−0.099
*∗∗*Correlation is significant at the 0.01 level (2-tailed).				

## Data Availability

By sharing the data included in our manuscript, we help in sharing knowledge with researchers in the wider scientific community.
